# Effects of a portion design plate on food group guideline adherence among hospital staff

**DOI:** 10.1017/jns.2017.60

**Published:** 2017-12-13

**Authors:** Dirk F. de Korne, Rahul Malhotra, Wai Yee Lim, Christine Ong, Ashu Sharma, Tai Kiat Tan, Thiam Chye Tan, Kee Chong Ng, Truls Østbye

**Affiliations:** 1 Medical Innovation & Care Transformation, KK Women's and Children's Hospital, Singapore; 2 Health Services & Systems Research, Duke-NUS Medical School, Singapore; 3 Erasmus School of Health Policy and Management, Erasmus University Rotterdam, Rotterdam, the Netherlands; 4 Health Services Innovation, University of Tasmania, Hobart, TAS 7000, Australia; 5 Nutrition & Dietetics, KK Women's and Children's Hospital, Singapore; 6 Sodexo Quality of Life Services, Singapore; 7 Patient Support Services, KK Women's and Children's Hospital, Singapore; 8 Office of Patient Experience, KK Women's and Children's Hospital, Singapore; 9 Department of Obstetrics & Gynaecology, KK Women's & Children's Hospital, Singapore

**Keywords:** Behaviour, Food groups, Guidelines, Adherence, T0, 2 months before the introduction of the design plate, T1, 1 month after the introduction of the design plate, T2, 3 months after the introduction of the design plate, T3, 6 months after the introduction of the design plate

## Abstract

Food group guideline adherence is vital to prevent obesity and diabetes. Various studies have demonstrated that environmental variables influence food intake behaviour. In the present study we examined the effect of a portion design plate with food group portion guidelines demarcated by coloured lines (ETE Plate™). A two-group quasi-experimental design was used to measure proportions of carbohydrate, vegetable and protein portions and user experience in a hospital staff lounge setting in Singapore. Lunch was served on the portion design plate before 12.15 hours. For comparison, a normal plate (without markings) was used after 12.15 hours. Changes in proportions of food groups from 2 months before the introduction of the design plate were analysed in a stratified sample at baseline (859 subjects, all on normal plates) to 1, 3 and 6 months after (in all 1016 subjects on the design plate, 968 subjects on the control plate). A total of 151 participants were asked about their experiences and opinions. Between-group comparisons were performed using *t* tests. Among those served on the portion design plate at 6 months after its introduction, the proportion of vegetables was 4·71 % (*P* < 0·001) higher and that of carbohydrates 2·83 % (*P* < 0·001) lower relative to the baseline. No significant change was found for proteins (−1·85 %). Over 6 months, we observed different change patterns between the different food group proportions. While participants were positive about the portion design plate, they did not think it would influence their personal behaviour. A portion design plate might stimulate food group guideline adherence among hospital staff and beyond.

Obesity is a leading cause of preventable death and disability globally, and contributes to hypertension, hyperlipidaemia, osteoarthritis, cancer, sleep apnoea and diabetes mellitus^(^^1^^)^. Diabetes mellitus is an increasing problem in both Western and non-Western countries, with the multi-ethnic South-East Asian city state Singapore being ranked second globally after the USA (10·53 and 10·75 %, respectively)^(^^2^^)^, and having the highest rate of diabetes-related hospital admissions in adults^(^^3^^)^.

Both excessive amounts and lack of variety in food intake are important determinants of diabetes and obesity. While healthy eating programmes often focus on food group category intake, food pyramids may be difficult to interpret^(^^4^^)^. Instead, increasingly ‘healthy plate’ pictorials indicating proportions for vegetables, proteins and staples are used, including the United States’ Healthy Eating Plate^(^^5^^)^, the UK's NHS Eat Well Guide^(^^6^^)^ and Singapore's ‘MyHealthyPlate’^(^^7^^)^.

Various studies conducted in both laboratory and restaurant settings have demonstrated that environmental variables influence food intake. Built environments influence the type and amount of food consumed on different levels. Borrowing from geographical paradigms, Sobal & Wansink^(^^8^^)^ distinguish four ubiquitous microscale built environments (or ‘scapes’) that are persistent but often unrecognised: kitchenscapes (availability, diversity, visibility of foods), tablescapes (variety, abundance and accessibility), platescapes (portion, package size, and arrangement and utensils type) and foodscapes (food-item forms and landmarks). These factors provide a subtle, pervasive and often unconscious influence on food intake, obesity and health. A meta-analysis of randomised controlled trials concluded that people consistently consume more foods when offered larger-sized tableware^(^^9^^–^^13^^)^.

Currently little is known about the potential influence of platescapes on food group intake. The effects of portion plates, where the recommended portions of vegetables, proteins and carbohydrates are printed on the actual plate, have only been studied as part of multicomponent interventions in small obese populations in Canada and the USA^(^^14^^–^^16^^)^. These studies showed that the combination of diet instructions sessions and a portion design plate seems to reduce weight. Bohnert *et al*.^(^^16^^)^ showed that sixteen African–American adolescent participants selected less food overall, more fruit, more broccoli *au gratin* and less steamed broccoli when using a portion plate. A small study in diabetics and obese Japanese confirmed these findings in a Asian setting^(^^17^^)^. However, as these four studies included multiple intervention components simultaneously (including teaching nutrition value and focus behaviour and lifestyle change), the effect of the plate design itself remains unclear. Moreover, one of the main potential benefits of plate design on dietary intake is its preventive function in normal populations.

Diet variations are large and platescapes vary between (and within) countries. The multi-ethnic city-state Singapore is known for its broad range of Chinese, Malay, Indian and Western food options. The Singapore Health Promotion Board (HPB) ‘healthy plate’ guidelines recommend proportions of 0·5 vegetables, 0·25 proteins and 0·25 carbohydrates per meal^(^^7^^)^. Adherence to these guidelines has not been studied. According to the latest national nutrition survey performed in 2010, only 11·2 % of adult Singapore residents consumed at least two servings of vegetables and fruits per d, compared with 14·3 % in 2004^(^^18^^)^. As a common circular plate can be divided into four equal parts, and roughly caters to four servings, two servings can be compared with half a plate (0·5 portions).

This study aims to assess the impact of a design plate on the relative proportions of food group intake. We hypothesised that use of the plate will result in better adherence to the Health Promotion Board Singapore (HPB) guidelines for carbohydrate, protein and vegetable intakes.

## Methods

**Fig. 1.**
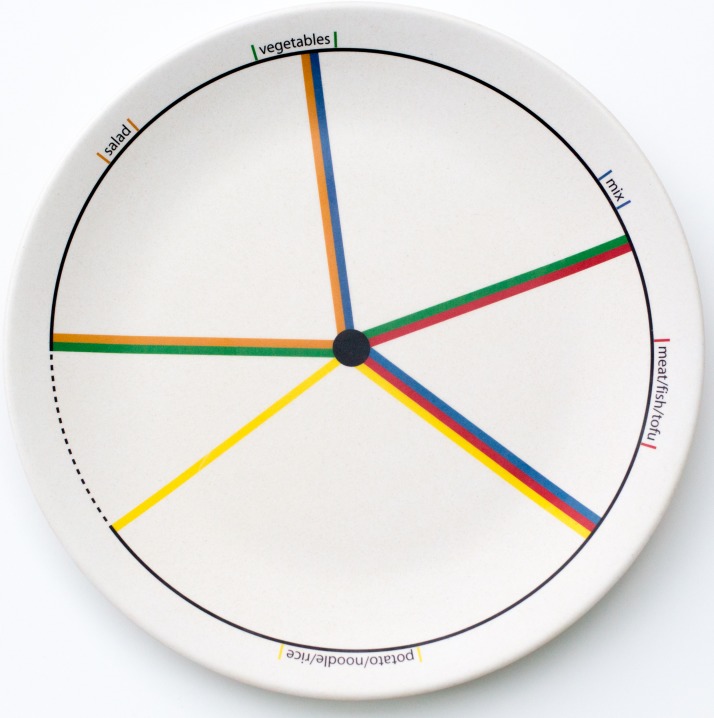
Design plate used in the study, with portion design allowing mixed food combinations.

The setting for the present study was a hospital staff lounge in Singapore where lunch is served to about 120 staff each day from a buffet-style counter. A two-group quasi-experimental design was applied. For 6 months, starting in October 2015, lunch was served on a design plate (ETE plate™, see Fig. 1)^(^^19^^)^, with portion guidelines printed in coloured lines indicating the various food groups to all staff who had lunch between 11.00 and 12.15 hours (design plate group). For comparison, a normal plate (with no markings) was used among all staff members who had lunch between 12.15 and 14.00 hours (normal plate group). Cashier receipt data from all staff taking lunch were included in the study. Paper leaflets with information about the design plate were available for all staff lounge users. The SingHealth Centralized Institutional Review Board exempted the study from review.

The lunch items offered at the buffet-style counter were: white rice, brown rice and potato (carbohydrates); chicken, fish, bean curd, dal and egg tofu (proteins); and Malay, Indian and Chinese vegetables.

### Intervention

We used an existing design plate (ETE plate™, see Fig. 1)^(^^19^^)^ with portion guidelines printed in coloured lines indicating the various food groups. This plate was found to cater to mixed portions of meat and vegetables, which is a common combination in the Asian food setting. The designed proportions are 0·440 for vegetables (including ‘salad’), marked in green; 0·160 for proteins, indicated on the plate as ‘meat/fish/tofu’, marked in red; 0·295 for carbohydrates, indicated on the plate as ‘potato/noodle/rice’ and marked in yellow. The combination of meat and vegetables is indicated as ‘mix’ and marked in blue. The plate also has a small (0·105) empty/miscellaneous area which serves to hold the thumb when walking from the buffet to the table, and, according to the designer, ‘reflects on all people whose plate cannot be filled’, indicated with a dotted line.

### Measures

The number of vegetable portions, protein (chicken, fish, bean curd, dal, egg tofu) portions, and carbohydrate (white rice, brown rice, potato) portions taken per individual plate were obtained from cashier receipt data, from baseline (T0, 2 months before the introduction of the design plate), to 1 month (T1), 3 months (T2) and 6 months (T3) after the introduction of the plate. As the food items served differed each day of the week but were usually similar for a particular day (for example, Chinese vegetables on Tuesdays), a sample of cashier receipts from five different weekdays spread over 5 weeks was analysed for T0 (September–9 October 2015), T1 (November–December 2015), T2 (February–March 2016) and T3 (June–July 2016). As the cashier receipt data showed the orders per individual food item ordered, we extracted the items and categorised them accordingly. Hospital staff ordered their preferred items from the buffet while a server held the plate and put the food on the plate. As the information was easily available from the same cashier receipt, we also recorded data on additional mixed or sliced fruits that were ordered within the same receipt.

We also surveyed a subset of staff lounge users at T1 and T3 to determine characteristics of the design plate and normal plate users, and understand their user experiences. Verbal informed consent was obtained. The surveys were distributed and collected during lunchtime. We asked the design plate users to indicate their opinions about the effect of the plate on the following five items: (a) eating more balanced; (b) eating more vegetables; (c) eating less proteins; (d) eating less staples; and (e) eating healthier. Agreement was self-recorded on a five-point Likert scale (1 = strongly disagree, 2 = disagree, 3 = uncertain, 4 = agree, 5 = strongly agree). The surveyors (research assistants) also requested qualitative feedback during collection in a standard way asking the same questions. Remarks were noted down verbatim.

### Data analysis

Analysis was done to assess changes in the proportions from baseline (T0; before introduction of the plate) to T1, T2 and T3 (after the introduction of the plate) for those who received the design plate, compared with the users of the normal control plate. Analysis was done to assess any changes in the proportions from baseline 3 and 6 months after the introduction of the plate.

We analysed the change in the ‘before 12.15 hours servings’ from before to after the introduction of the design plate, and the change in the ‘after 12.15 hours servings’, in mean proportions of each food group, and compare the extent to which these changes differ from each other. We first analysed the data by combining all three after observations, secondarily considering the change from T0 to T1, from T0 to T2 and from T0 to T3 separately. Comparisons were performed using *t* tests, using Stata version 13.1 (StataCorp LLC), two-tailed at the 5 % significance level.

## Results

### Participants

Hospital staff characteristics were taken from the user experience survey, which included a subset of staff lounge users at T1 (*n* 76) and T3 (*n* 75). Three-quarters of the participants were female. The mean age was 32 (range 20–58) years old. The users had nursing, medical, allied health or administrative backgrounds. The participants had Chinese, Malay, Indian and Filipino ethnicity. The characteristics of the users before 12.15 hours did not differ from the users after 12.15 hours (see Table 1).

**Table 1.** Characteristics of hospital staff lounge users in the design plate and normal plate groups, based on the subset survey (Percentages; mean values and ranges)

	Design plate	Normal plate	P*
Number of participants	114	91	
Response rate	96	89	
Female	75·5	69·8	0·38
Age (years)			0·01
Mean	32	36	
Range	20–58	19–60	
Race			0·43
Chinese	58·5	63·1	
Malay	10·4	14·3	
Indian	15·1	7·1	
Filipino	13·2	13·1	
Others	2·8	2·4	
Designation			0·17
Nursing	35·1	19·5	
Medical	16·7	19·5	
Allied health	18·5	19·5	
Administrative	9·3	24·4	
Others	20·4	17·1	

* χ^2^ Test.

### Guideline adherence

Analysis was done to assess any changes in the proportions from baseline (T0, *n* 442 meals, before 12.15 hours; *n* 416 meals after 12.15 hours, all on normal plates) to 1 month (T1, *n* 395 on design plates, *n* 373 on normal plates), 3 months (T2, *n* 355 on design plates, *n* 296 on normal plates) and 6 months (T3, *n* 266 on design plates, *n* 299 on normal plates) after the introduction of the plate.

After introduction of the design plate, guideline adherence in the design plate users had significantly improved for vegetables (+4·71 %; before (T0_normal_): 0·317 (sd 0·184), after (T1_design_+T2_design_ + T3_design_): 0·364 (sd 0·172); *P* < 0·001) and carbohydrates (−2·83 %; before (T0_normal_): 0·351 (sd 0·135), after (T1_design_ + T2_design_ + T3_design_): 0·323 (sd 0·104); *P* < 0·001). No significant improvement (−1·85 %; before (T0_normal_): 0·332 (sd 0·160), after (T1_design_ + T2_design_ + T3_design_): 0·313 (sd 0·145); *P* = ·02) was found for proteins. When comparing these outcomes with the observed changes in the normal plate group, the differences for vegetables and proteins were 4·20 and 4·56 %, respectively, while for carbohydrates the reduction in the normal plate group was 0·39 % larger (see Table 2).

**Table 2.** Changes in food group proportions after introduction of the design plate (Mean values and standard deviations)

		Before introduction of the design plate (T0)				After introduction of the design plate (T1 + T2 + T3)						
		Before 12.15 hours		After 12.15 hours		Before 12.15 hours		After 12.15 hours				
		Normal plate		Normal plate		Design plate		Normal plate		Difference in design plate	Difference in normal plate	Overall difference for
	HPB guidelines	Mean	sd	Mean	sd	Mean	sd	Mean	sd	(%)	(%)	both plates (%)
*n*		442		416		1016		968				
Carbohydrates	0·250	0·351	0·135	0·353	0·142	0·323	0·104	0·321	0·108	−2·8*	−3·22	−0·39
Vegetables	0·500	0·317	0·184	0·352	0·187	0·364	0·172	0·357	0·174	+4·7*	+0·51	4·20
Proteins	0·250	0·332	0·160	0·295	0·166	0·313	0·145	0·322	0·161	−1·85	+2·71	4·56

HPB, Health Promotion Board, Singapore.

* Significant (*P* < 0·001).

**Fig. 2.**
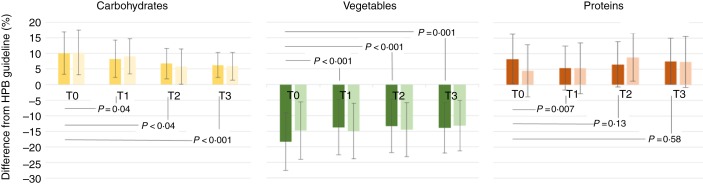
Differences between Health Promotion Board, Singapore (HPB) guidelines and food group proportions measured, before (T0), *v*. 1 month (T1), 3 months (T2) and 6 months (T3) after introduction of the design plate. In all three graphs, the dark colours refer to the design plate group, and the lighter-coloured bars refer to the normal plate group. Values are means, with standard deviations represented by vertical bars. Between-group comparisons were performed using *t* tests.

Over the 6-month period, we observed different change patterns between the different food group proportions (see Fig. 20. Increase in the vegetable proportion was observed in the first month (*P* < 0·001), and stayed on a similar level for the subsequent months. Carbohydrate proportions were reduced step-wise over the 6 months. While protein proportion showed an initial decrease (*P* = 0·007), no significant change was observed for the subsequent months and the overall period. However, similar direction and magnitude of change in proportions were also observed for the eaters after 12.15 hours (see Fig. 2).

Additionally, when comparing the differences in fruit orders (in addition to the food on the plate) we observed an increase of 2·0 % (before: 16·7 %, after: 18·7 %) in the design plate group, and an increase of 5·2 % (before: 6·7 %, after: 11·9 %) in the normal plate group. No differences were observed in brown rice orders (*v*. white rice) in the carbohydrate category, or fish orders (*v*. chicken) in the protein category.

### User experiences

The staff lounge users were positive about the design of the plate and the awareness that it creates for healthy eating. However, they did not think the plate influenced their own choices. For all five items, the scores were below the scale centre (see Table 3). Highest scores were obtained for ‘The design plate helps me to eat more balanced’ (mean 2·58 (sd 1·01)), and lowest for ‘The design plate helps me to eat less proteins’ (mean 1·98 (sd 1·04)) and ‘less staples’ (average 1·98 (sd 1·04)). While the scores were higher after 6 months compared with 1 month in both groups, the differences were not significant.

**Table 3.** Staff lounge users’ opinions on potential functions of the design plate, at 1 month (T1) and 6 months (T3) after implementation of the design plate (Mean values and standard deviations)

	T1 (after 1 month) (*n* 76)		T3 (after 6 months) (*n* 75)		
The design plate helps me to eat	Mean	sd	Mean	sd	P*
1. More balanced	2·30	0·94	2·58	1·01	0·07
2. More vegetables	2·27	1·04	2·42	1·00	0·74
3. Healthier	2·31	1·04	2·42	1·04	0·63
4. Less staples	1·98	1·04	2·28	1·00	0·23
5. Less proteins	2·08	1·06	1·98	1·04	0·56

* Two-sample *t* test; 1 (strongly disagree)–5 (strongly agree) Likert scale.

## Discussion

Our data indicate that a portion design plate might stimulate food group guideline adherence. At 6 months after introduction of the design plate, guideline adherence had significantly increased for vegetables and carbohydrates. These findings confirm the earlier studies done in small obese populations^(^^14^^–^^16^^)^ and indicate that a portion design plate might be relevant for larger and still healthy populations and potentially could have a disease-preventive function. The increase in vegetable proportion is promising as lack of vegetables and fruit intake is prevalent in many countries, including Singapore. While the latest national nutrition survey was only performed 7 years ago in 2010, we have no indications that the percentage of 11·2 % of adult Singapore residents consuming at least two servings of vegetables and fruits (roughly equivalent to half a plate) per d has increased by now^(^^18^^)^.

The decrease in carbohydrates was not expected as we expected in line with common observations in Asia that all eaters would continue to take a portion of rice anyway. The respondents surveyed shared a similar opinion, indicating the lowest expectations for an effect on carbohydrates. However, during our study period the Singapore health authorities sparked a nationwide debate as part of their declared ‘war on diabetes’, indicating the relationship between white rice intake and diabetes based on a Harvard 2012 meta-analysis^(^^20^^)^ and resulted in local press headlines such as ‘The rice you eat is worse than sugary drinks’^(^^21^^)^. It might be possible that this and other ongoing health campaigns have influenced the present study as we did observe a similar direction and magnitude of change in proportions for the users of the normal plate. As the participants were hospital staff, they might potentially have a higher health consciousness compared with a sample from the general population.

Knowing the various cultural tastes that the plate needs to cater to, the marking of lines on the plate allows for flexibility. For example, the ‘mix’ portion in between the blue lines (see Fig. 1) allows for favourite Singaporean meals where vegetables and meat are premixed. Therefore we have not chosen a more rigid plate design with separate sections or boxes while these were also available. An interesting insight during the set-up of the study was the fact that the cashier labelling for some of the items was related to the wrong food groups. Potatoes and dal were considered to be vegetables and hence labelled by the cashier as such. Before we could commence the study proper, the display of the cashier was redesigned. We recognise more issues with regard to the design of our real-world study. While we have designed a quasi-experiment, it was easily possible that the same eaters were part of the design plate group on one day (having lunch before 12.15 hours) and being part of the normal plate group another day (having lunch after 12.15 hours). This might have resulted in contamination between the two groups.

In most lunch catering facilities in Asia, the eaters are not self-serviced; it is the plates that are served. In the present study setting, the eaters pointed to the food that was displayed in a buffet-style setting, but the server positioned the actual food on the plate. As the eaters are confronted with the printed guidelines during eating for a longer period (eaters are using the staff lounge regularly), actual effects can only be expected after he has been exposed to the plate one or more times, and consequently based on the confrontation with the guidelines during eating will change his order behaviour. However, there might have been bias with this regard and a more controlled study design, including direct observation of the amount size (using, for example, photographs or video stills) as well as follow-up in the same participant, is necessary to further validate the findings.

We did observe a decrease of proteins; however, this was not significant. Meat is considered the main part of food in many parts of Asia. The study hospital caterer indicated during the study that the number 1 complaints in eateries is clients who want more meat or think they receive too much rice instead of meat or vegetables. As the present study only focused on the meal taken for lunch, we do not have insights in the food group proportions taken on other moments of the day.

While participants were positive about the portion design plate, they did not indicate it to influence their personal behaviour. This confirms that design and ‘scapes’ do influence users without them being aware. Design thinking and behavioural nudging play a pivotal role in order to improve guideline improvement^(^^2^,^3^^–^^2^,^6^^)^. Therefore a portion design plate might potentially be powerful in changing eating behaviour and cost-effectively preventing obesity and diabetes.

Changing the environmental and micro ‘scape’ influences related to eating will be essential to increase food group guideline adherence. Earlier studies have shown that portion plates might be more effective in reducing weight in obese adults compared with standard dietary teaching strategies^(^^14^^,^^2^,^7^^,^^2^,^8^^)^. Smartly designed portion plates that move beyond local eating habits and preferences might influence eaters’ behaviour on an even large scale. Follow-up studies with a more controlled research design including health outcomes of individual plate users will be important to further validate the role of plate design as a comprehensive effective and efficient prevention strategy for obesity and diabetes.
